# LINGO-1 is a New Therapy Target and Biomarker for Ewing Sarcoma

**Published:** 2017-01-05

**Authors:** Arvind Jain, Jing Zhang, Terence Rabbitts

**Affiliations:** Weatherall Institute of Molecular Medicine, University of Oxford, Oxford OX3 9DS, UK

**Keywords:** Ewing sarcoma, Chromosomal translocation, Antibody, Single domains, iDAb, LINGO-1, ADC, Nanoparticles

## Abstract

Ewing sarcoma is a predominantly paediatric cancer with a high rate of metastasis and reoccurrence. A new surface marker called LINGO-1 was recently identified on Ewing tumours as a potential target for antibody-mediated therapies. However, such targeting requires caution because of LINGO-1 expression on some brain cells. Although the blood-brain-barrier exists, small amounts of antibody may cross this barrier and cause harmful side-effects. In this perspective, we suggest some options to alleviate this risk that can make targeting tumour cells expressing the LINGO-1 antigen a safe option.

## Ewing Sarcoma Occurrence, Current Treatment and Prognosis

Ewing sarcoma is rare bone cancer, first described by James Ewing in 1921 as endothelioma. It is poorly differentiated small round tumour with high rate of metastasis and relapse. Ewing sarcoma (EWS) is the second most common bone cancer after osteosarcoma affecting children, with a median age of 15 years old and occurs more frequently in males (with a ratio of 1.5:1). Bones are primary site of EWS but may also occur in the soft tissues. In rare occasions it can be of extra osseous origin, including paravertebral spaces, arms, legs, head and neck, skin and chest wall. Primary EWS sites include pelvis (26%), long bones (47%) chest wall (16%) and spine (6%). Advanced EWS with metastasis and disseminated disease is found in around 25% of cases during diagnosis. The primary sites of the metastasis are lungs, followed by distal bones and bone marrow [1–3].

All EWS have a chromosomal translocation involving the *EWSR1* gene on chromosome 11 band q12.2 or the related *FUS* gene (chromosome 16, band p11) and more than 85% of EWS exhibit the chromosomal translocation between chromosome 11 and 22 t(11; 22) (q24.3:q12.2) resulting fusion of ESWR1 gene with *FLI1* gene (Friend leukaemia virus integration site). FLI1 is a member of ETS family of transcription factors. Other gene arrangements causing sarcoma includes *EWSR1-ERG* fusion t(21;22) (q22:q12); *EWSR1-ETV1* fusion t(7;22)(p22;q12); *EWSR1-E1AF* fusion t(17,22) (q12;q12); *EWSR1-FEV* fusion t(2;22) (q33;q12) also *FUS-ERG* and *FUS-FEV* reviewed in [4].

Standard treatment for the patients with EWS includes a multimodal therapy where chemotherapy is employed to reduce the tumour followed by local management by surgical excision and/or radiotherapy, depending upon the circumstances. Recent advancements in the multimodal therapy have improved the outcome with the localised Ewing sarcoma to 70% 5-year survival. The management of advanced stages with the metastasis and relapse still remains the challenging, having less than 30% 5-year survival [5]. Further, current treatments are associated with cumulative and late toxicities. Chemotherapy has very limited responsive rate as second or third-line treatment for the relapsed EWS, causing cumulative haematological and neurological toxicity with lower response rate [6]. These facts indicate a requirement of an efficient and safe advanced therapy for the management of EWS that can provide longer and a better quality of life.

## New Options for EWS Antibody-Based Therapy

The major underlying reason for the low efficiency and high toxicity of chemotherapy is the limited availability of chemotherapeutic agents to the target cancer site and their high re-distribution throughout the body after systemic administration, where they exert unwanted toxic effects. It has been reported that the combinatorial system and high doses of drugs can be more effective in the management of the metastases, but are associated with prominent side effects. Application of advanced drug delivery strategies that can provide exclusive and safe delivery to the cancer site can address these issues. The concept of “Magic Bullets” was proposed by Paul Ehrlich a century ago, and a variety of targeted delivery options have been developed and explored. Among those antibody-based strategies have been most impressive as antibodies offer a high level of specificity and can be generated against all protein targets. Furthermore, antibodies that bind to tumour cell surface antigens can carry therapeutic cargos that are attached to the antibody, delivering to local tumour sites. Two types of antibody-mediated therapy could be applied to EWS treatment, viz. antibody drug conjugates (ADCs) and immuno-nanoparticles (i-NPs) carrying drugs. For both, specific cell surface markers are ideal to give specific tumour localization.

In the case of ADC, potent cytotoxic drugs chemically linked to a specific anti-tumour antibody, can allow selective delivery directly into cancer cells, while leaving healthy cells unaffected. Small molecule drugs for conventional chemotherapy are designed to eliminate cancer cells, however they also harm healthy cells. The ADC that combines the cell killing activity of chemotherapy drug and anti-tumour selectivity of antibody achieves targeted delivery as well as efficient cell killing by increasing the local drug concentration at the tumour site [7].

i-NPs are colloidal systems of the nanometer size that are decorated with a specific antibody on their surface and carry the cargo in the form of drug, protein/peptide or nucleic acid. Various polymers, lipids, metal and protein-based nanoparticles have been developed that are assisted by surface antibody to recognize the target cancer site for the specific delivery of the payload. This would help to achieve the higher concentration of the chemotherapeutic agent at the cancer site for enhanced drug efficiency as well as sparing healthy cells. In addition, these vehicles can potentially be optimized in their physiological properties to reach the more distal locations of the body [8,9].

## LINGO-1 is a New Marker Expressed on Ewing Sarcoma Tumour Surface

**Figure 1:**
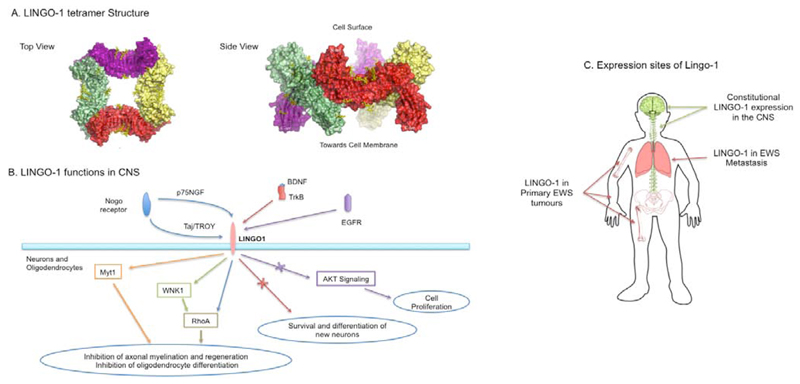
LINGO-1 structure, function and expression sites. A. Crystal structure of LINGO-1 tetrameric extracellular domain shows four LINGO-1 oligomers (depicted in yellow, green red and magenta) in a ring like structure on cell surface (adopted from Mosyak et al. [13]). B. Role of LINGO-1 is responsible for neuronal and oligodendrocytes growth and regeneration pathways. C. Outline of LINGO-1 expression locations in the CNS and in EWS tumours.

As first objective to develop targeted therapy, a unique recognition site is required on the surface of the EWS cancer cells against which antibody or immuno-nanoparticles can be programmed for the delivery. In our quest to identify tumour-associated antigens, we have developed a strategy to indirectly study the cell surface of cells using next generation sequencing RNA-seq data as a surrogate to interrogate cell surface molecules (herein designated as the surfaceome) [10]. Applying method to study Ewing sarcoma cells, we found candidate mRNA species encoding EWS-associated surface proteins (included Ig-like domain proteins, G protein-coupled receptor, ion channels and ion transporters) and in particular observed a unique protein LINGO-1 that could be exploited to distinguish EWS cells from normal cells. The *LINGO-1* cDNA was originally discovered by Carim-Todd et al. [11], and called LERN-1, encoding a leucine-rich repeat and immunoglobulin domain-containing protein that has the advantage of having an accessible extracellular domain (Figure 1). It is a potent negative regulator for various CNS related functions such as neuronal differentiation and growth, oligodendrocyte myelination, axon branching and regeneration (Figure 1). Thus, LINGO-1 signalling has implications in various neuro-pathological conditions such as Parkinson’s disease, schizophrenia, Alzheimer’s disease, multiple sclerosis, anxiety etc [12].

## LINGO-1 Antibodies for Possible Ewing Sarcoma Treatment

When we studied the surface of EWS tumour cells, we discovered the cell surface protein LINGO-1 is highly expressed and normal expression seems to be restricted to CNS in post-natal tissues [10]. Our initial studies have shown that anti-LINGO-1 antibodies are specifically recognised by EWS tumour cells and, following binding to LINGO-1, the antibody becomes internalized to be localized to early endosomes and lysomsomes (suggesting that LINGO-1 is internalised via the endosome-pathway). These results reveal the potential of LINGO-1 as a therapeutic target for a wide range of ADC or immuotoxin treatments on EWS. Further, ADC experiments showed that EWS tumour cells are killed by anti-LINGO-1 ADC whereas mesenchymal stem cells, that lack LINGO-1 expression, survive [13]. Drug delivery for cytotoxicity of EWS tumours is therefore an option for EWS therapy but CNS toxicity with such ADC use remains a major concern (see next section concerning the blood brain barrier).

## LINGO-1 and the Blood-Brain Barrier

LINGO-1 is a possible target for EWS because it has a very restricted post-natal expression [14]. However, the protein is expressed in the brain, which is separated from the blood stream by blood brain barrier (BBB). The BBB is an interface between the CNS and peripheral blood circulation achieved by the tight junctions of endothelial cells that limit the transport of water-soluble components from systemic circulation to the brain, including circulating antibodies [15]. Although, most antibodies do not penetrate the brain, there is a low level transfer and this could be a source of major side effects if anti-LINGO-1 antibodies are used therapeutically (particularly as ADCs). To obviate this, specifically designed drug delivery systems would be needed that cannot cross the and thus render LINGO-1 programmed drug delivery systems exclusive to the EWS tumours with no potential CNS toxicities.

## Bispecific Monoclonal Antibodies

It may be possible to overcome this by two approaches, namely bispecific monoclonal antibodies (bsMAb) or i-NPs. Many different bsMAb formats have been developed for combining specificities and functions of two antibodies targeting different antigens. Many of them are currently in different clinical development stages with Removab^®^ and Blincyto^®^ having been approved for therapy. While some bsMAbs are designed to recruit and activate the T cells upon binding to the target cells (so-called bispecific T cell engagers, BiTEs [16,17], most others are designed to dual targeting two disease mediators in order to enhance tumour specificity and potency as well as overcome biologic redundancy [18]. A good candidate for the second target molecule for an anti-LINGO-1 bsMAb in EWS is CD99. This molecule is a glycosylated protein associated with EWS although it is not EWS-specific and the expression of CD99 could be also found on leukocytes [19]. Alternatively, other EWS-specific surfaceome that were found in our RNA-seq analysis [10] and these offer options for bsMAbs together with anti-LINGO-1 binding. More investigation of these candidates is important for the possible bsMAb development approach.

## Immuno-nanoparticles (i-NPs) and PEGylation

**Figure 2:**
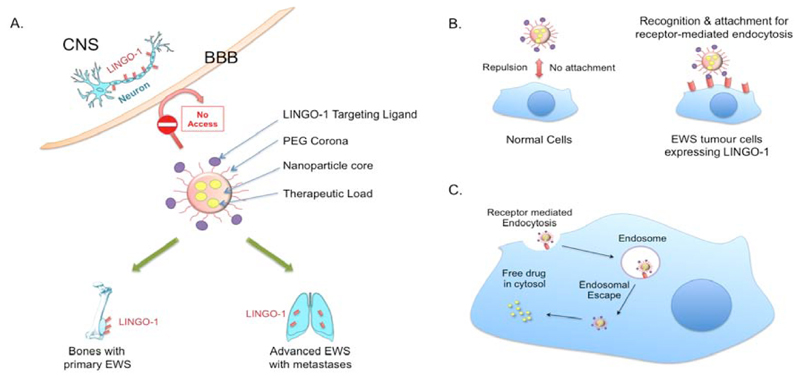
Hypothetical advanced targeted drug delivery system for Ewing sarcoma. A. LINGO-1 is expressed on cell of the CNS protected by the BBB. The presence of a biomimetic corona around i-NPs prevents their crossing the BBB to avoid any potential CNS side effects. An additional advantage of using i-NP carrying anti-cancer drugs would be prolonged blood circulation half-life. B. The anti-LINGO-1 i-NPs would only recognise EWS tumour cells with LINGO-1 for adhesion. C. After binding, LINGO-1 cause’s receptor-mediated endocytosis followed by NP disruption of the endosome for release their drug contents.

Another potential route of LINGO1-directed drug delivery is therefore using i-NPs that are coated with anti-LINGO-1 antibody and carry therapeutic drugs as these will not cross the BBB unless have been specifically designed for this purpose [20]. Figure 2 outlines this situation. PEGylated drug delivery systems such as PEGylated antibodies have proven efficiency for the targeted systemic delivery to the cancer sites. Utilizing a PEG corona around the delivery NP would also impart biomimetic properties yielding higher blood circulation half-life [21]. Delivery systems in the nanometer size range have added advantage of enhanced delivery to cancer sites due to the EPR effect of enhanced permeability due to the fenestrated vasculature and retention after penetration [22]. For bone and lung specific delivery, various nanoparticles have been developed that can be classified as organic nanoparticles, includes poly (lactic-co-glycolic), acid based polymeric NPs, albumin NPs and liposomes [23]. Some or all of these have potential to be guided to tumour locations by anti-LINGO-1 antibody (or antibody fragment) coating.

## Drugs and Macrodrugs for EWS Therapy

Apart from chemical drugs nanoparticles can be used to deliver the new generation of sophisticated bio-therapeutic agents (such as antibody single domains, nanobodies) and nucleic acid based treatments (such as siRNA, mRNA, shRNA) that cannot be systemically used *per se* due to the inability to enter into cells. This new generation of macromolecular drugs (referred to as macrodrugs to distinguish them from conventional chemical drugs) is being developed that can potentially replace or augment current drug therapies. One important feature of these macrodrugs is that they are unlikely to give rise to drug resistance as can happen with small molecule drugs. Therefore, i-NPs can become an important part of cancer therapy. In the case of EWS, the LINGO-1 surface marker can be the target molecule for delivery of macrodrug cargos.

## Conclusion

There are three main areas where the surfaceome can be crucial in any cancer treatment. First, tumour-associated proteins can be useful in antibody-based diagnosis and LINGO-1 should be important to avoid mis-diagnosis in cases that present with unusual chromosomal translocations that are different from the more consistent translocation hallmarks such as the t (11;2). The second use of is in specifying cancer cells over normal counterparts for cargo delivery. EWS-expressed LINGO-1 can provide a target on afflicted cells for antibody drug conjugates in this role. The third area of important is to fulfill a loading depot role for receipt of immuno-nanoparticles carrying drug cargos for internalization into tumour cells. In Ewing sarcoma, all these options have promise using the LINGO-1 surface antigen. Strategic development of LINGO-1 targeted delivery vehicles can deliver drugs or macrodrugs specifically to EWS tumour cells sparing the normal body tissues. LINGO-1, recently reported to be specifically expressed over the EWS cells, thus offers a unique opportunity to achieve new, safer therapy.
